# Night work and breast cancer risk in a cohort of female healthcare employees in Stockholm, Sweden

**DOI:** 10.1136/oemed-2022-108673

**Published:** 2023-05-03

**Authors:** Per Gustavsson, Carolina Bigert, Tomas Andersson, Manzur Kader, Mikko Härmä, Jenny Selander, Theo Bodin, Maria Albin

**Affiliations:** 1 Institute of Environmental Medicine, Karolinska Institutet, Stockholm, Sweden; 2 Centre for Occupational and Environmental Medicine, Region Stockholm, Stockholm, Sweden; 3 Work Ability and Working Careers, Finnish Institute of Occupational Health, Helsinki, Finland

**Keywords:** Cancer, Epidemiology, Occupational Health, Health Personnel, Shift Work Schedule

## Abstract

**Objectives:**

Night work has been classified as probably carcinogenic to humans by the International Agency for Research on Cancer, but epidemiological evidence was considered limited due to variability in findings and potential bias. This study aimed to investigate the risk of breast cancer in a cohort with detailed and registry-based data on night work.

**Methods:**

The cohort comprised 25 585 women (nurses and nursing assistants) employed 1 year or more between 2008 and 2016 in the healthcare sector in Stockholm. Information on work schedules was obtained from employment records. Breast cancer cases were identified from the national cancer register. HRs were estimated by a discrete time proportional hazards model, adjusting for age, country of birth, profession and childbirth.

**Results:**

There were 299 cases of breast cancer, 147 in premenopausal and 152 in postmenopausal women. The adjusted HR of postmenopausal breast cancer in association with ever versus never working nights was 1.31 (95% CI 0.91 to 1.85). Eight or more years of night work was associated with an increased risk of postmenopausal breast cancer, HR=4.33 (95% CI 1.45 to 10.57), based on five cases only, though.

**Conclusions:**

This study is limited by a short period of follow-up and a lack of information on night work before 2008. Most exposure metrics showed no association with breast cancer risk, but there was an elevated risk of postmenopausal breast cancer in women after 8 or more years of night work.

**WHAT IS ALREADY KNOWN ON THIS TOPIC**

- Night shift work has been classified as probably carcinogenic to humans by the International Agency for Research on Cancer. The epidemiological evidence was considered limited due to variability in findings, potential bias and the quality of the exposure assessment in some of the studies.

**WHAT THIS STUDY ADDS**

- This study was based on detailed data on work schedules obtained from employment records and showed an elevated risk of breast cancer in postmenopausal women having worked at night for 8 years or more. However, the conclusions are limited due to a short period of follow-up and lack of information of night work before 2008.

**HOW THIS STUDY MIGHT AFFECT RESEARCH, PRACTICE OR POLICY**

- This study gives some support to the evidence for an association between night work and breast cancer. A prolonged follow-up will give a more precise conclusion from this study.

## Background

Breast cancer is the most common cancer among women, causing over 2 million deaths worldwide in 2019.^1^ The most prominent risk factors in the population are related to reproduction. The risk seems to be positively related to internal or external exposure to oestrogen, related to factors like late or no childbirths, early menarche, late menopause or hormone replacement therapy. Well-established external risk factors include alcohol consumption and exposure to ionising radiation.^2^

Studies on breast cancer aetiology and occupational exposure have during the last two decades been focused on the role of night and shift work. Work at night is hypothesised to be associated with an increased risk of breast cancer due to disruption of the circadian rhythm.^3^ Exposure to light at night has been suggested as one potential mechanism since light at night suppresses the normal nocturnal peak of the hormone melatonin, which has antitumourigenic properties.^4^

The International Agency for Research on Cancer (IARC) has evaluated the evidence for a cancer hazard from exposure to shift or night work at two occasions, most recently in 2019.^3^ On the basis of sufficient evidence in animals, strong mechanistic evidence and limited evidence in humans, the IARC classified night shift work as probably carcinogenic to humans (group 2A). There were several high-quality studies, predominantly of case–control design, which supported a causal association with breast cancer, but several cohort studies found no such evidence. The working group considered the human epidemiological evidence as limited, in view of the variability between studies and methodological problems, partly related to differences in exposure assessment quality.^5^ Especially, there has been a lack of cohort studies with sufficiently detailed exposure assessment. Studies published after the IARC evaluation have also shown variable results. A cohort study of female Norwegian offshore petroleum workers showed no evidence of an association between night shift work and breast cancer,^6^ while a cohort study of female Finnish public sector workers showed an increased risk of breast cancer in association with shift work with and without nights in women aged 50+ after 10 years or more of follow-up.^7^ A case–control study from Poland showed a positive association between night work and breast cancer,^8^ and a large cohort study from the USA, the ‘Sister study’ found little evidence for an association between rotating shift work or work at night and breast cancer incidence.^9^

This study aimed to investigate the risk of breast cancer in association with night work in a cohort with individual and very detailed registry-based data on work schedules.

## Methods

The cohort comprised healthcare workers employed by Region Stockholm (formerly Stockholm County Council) for at least 1 year from 1 January 2008 to 31 December, 2016. Details on the formation of the cohort have been published earlier.^10^ Briefly, the cohort was identified from a computerised employment register, HEROMA and comprised workers in occupations often involving night and shift work, that is, nurses and specialist nurses (including midwives), nursing assistant and occupations like caregivers, accommodation assistants and personal assistants. Staff were included both from hospitals and outpatient clinics. Information on employment or work schedules was not available before 2008, even if some of the cohort members may have been employed earlier. Physicians were not included since working hours were not recorded at the same level of detail for this group.

Working hours in healthcare in Sweden is typically organised in the following way: Day-time workers start at 7:00 or 8:00 and work 8 hours. Afternoon shifts start at 14:00 and end at 21:00 hours. Night shifts start at 21:00 and end at 7:00 hours.

Information on working schedules hour by hour from 1 January, 2008 up to 31 December, 2016 was obtained from the HEROMA system. These data were used to define start and end of work shifts, which in turn were classified as (A) a night shift (≥3 hours of work between 22:00 and 06:00 hours or (B) a non-night shift, which could be either a daytime shift (starts after 06:00 and ends no later than 18:00 hours), or an afternoon shift (starts after 12:00 and ends later than 18:00 hours but not a night shift). The definition of night work and shift types follows closely to the definition suggested by IARC Monograph Volume 124: Night Shift Work (at least 3 hours between 23:00 and 06:00 hours),^3^ but has been updated to the Swedish law (at least 3 hours between 22:00 and 06:00 hours) and a study from Finland.^11^ The cohort subjects were then classified dynamically, for each year of follow-up, regarding any previous night-work (yes/no), the total number of nights worked, and the number of years with any night work. The latter variable was classified in four predefined classes, 0, 1–3, 4–7 and 8+ years. Eight or more years of night work was selected as the highest category since this gave 2 years of observation in the highest class, considering that the maximum duration of night work was 9 years (2008–2016).

First time diagnoses of breast cancer (International Classification of Diseases, Tenth Revision = C50) were obtained from the Swedish National Cancer Registry. Information on vital status, country of birth and emigration were obtained from Statistics Sweden, and information on childbirths (back to 1973) was obtained from the National Medical Birth Registry. Data were linked by the civil registration number, unique to every Swedish resident.

The risk of breast cancer in association with night work was investigated by a discrete time proportional hazards model, stratifying the follow-up period in calendar years.^12^ The analysis was adjusted for the time-dependent variables age and childbirth (yes/no, before the year of observation), and the fixed variables profession and country of birth. Adjustment for calendar year of follow-up was inherent in the analytical model. Stratification for menopausal status was based on attained age 55, based on an earlier prospective cohort study of Swedish women.^13^ Risk calculation started the year after 1 year of employment and ended at the first occurrence of breast cancer, emigration, death or the end of follow-up (31 December 2016), whichever occurred first. Test for trend was based on inclusion of total number of years of night work, or number of night shifts/100 as continuous and time-dependent variables in the model. The latter two trend-test models were also additionally adjusted for any night work, to disentangle the potential effect of working night as such and the quantity of night work. The rationale for this is that those who work nights may be different from those who do not in some respect with relevance to breast cancer risk.

We used SAS software V.9.4 for Windows (SAS Institute) for all statistical analyses.

## Results

The cohort comprised 25 585 women, 58% were registered nurses, including midwives, and 42% were nursing assistants or had corresponding occupations (practical nurses), (table 1). At the end of follow-up, 39% of the cohort members had ever worked at night. About 55% of the cohort were under age 50 at beginning of follow-up, and among those who had ever worked at nights 65% were under age 50. Those who had ever worked at night were younger than those who had not. Having worked nights was more common among nursing assistants than among nurses, but country of birth differed very little between the two groups. Ever having given birth to a child was slightly more common among those who had not worked at nights than among those who had.

**Table 1:** Characteristics of the study population

	Full cohort		Never worked at nights*		Ever worked at nights*	
	N	%	N	%	N	%
**All**	25 585	100.0	15 675	61.3	9 910	38.7
Age†						
<40	7 629	29.8	3 881	24.8	3 748	37.8
40–49	6 358	24.9	3 705	23.6	2 653	26.8
50+	11 598	45.3	8 089	51.6	3 509	35.4
Country of birth						
Sweden	20 334	79.5	12 606	80.4	7 728	78.0
Nordic countries except Sweden	1 546	6.0	971	6.2	575	5.8
Europe except Nordic countries	1 228	4.8	685	4.4	543	5.5
Outside Europe	2 475	9.7	1 413	9.0	1 062	10.7
Profession						
Nurses including midwives	14 977	58.5	8 821	56.3	6 156	62.1
Nursing assistants, others	10 608	41.5	6 854	43.7	3 754	37.9
Childbirth, yes*	19 148	74.8	12 025	76.7	7 123	71.9

*At end of follow-up.

†At beginning of follow-up.

The cohort comprised 160 200 person-years of observation during the period 2009–2016 (table 2). The maximum duration of time under risk was 8 years, and the maximum duration of recorded night work was 9 years (2008–2016). The number of person-years per exposure category is presented in tables 2 and 3. There were 299 cases of first-time breast cancer in the cohort, 147 in premenopausal and 152 in postmenopausal women. Ever having worked at night was not associated with the risk of breast cancer (table 2). More than 8 years of night work was associated with an increased unadjusted HR of 3.43 (95% CI 1.18 to 7.95), based on five cases only, though. The adjusted HR was non-significantly elevated, 2.80 (95% CI 0.96 to 6.52). Using number of night years as a continuous variable in the model showed no evidence of an association with breast cancer risk, and neither did inclusion of a variable reflecting number of nights. The two latter models were further modified by inclusion of an indicator for ever having worked at night, but this did not alter the results.

**Table 2:** Risk of breast cancer in association with various aspects of night work in six separate models

	Person-years	Cases	Crude HR	Adjusted HR*
No night work	107 203	216	1	1
Night work ever	52 997	83	0.77 (0.59–0.99)	0.96 (0.74–1.23)
No night work	107 203	216	1	1
1–3 years night work	36 472	57	0.78 (0.58–1.04)	1.08 (0.80–1.44)
4–7 years night work	15 707	21	0.63 (0.39–0.97)	0.65 (0.40–1.00)
8+ years night work	818	5	3.43 (1.18–7.95)	2.80 (0.96–6.52)
No night work	107 203	216	1	1
Risk per year of night work	52 997	83	0.96 (0.89–1.03)	0.98 (0.92–1.05)
No night work	107 203	216	1	1
Night work ever†	52 997	83	0.64 (0.40–0.98)	1.04 (0.66–1.62)
Risk per year of night work			1.07 (0.95–1.20)	0.97 (0.86–1.09)
No night work	107 203	216	1	1
Risk per 100 nights	52 997	83	1.00 (0.92–1.07)	0.98 (0.91–1.05)
No night work	107 203	216	1	1
Night work ever†	52 997	83	0.69 (0.50–0.93)	1.01 (0.72–1.39)
Risk per 100 nights			1.06 (0.97–1.15)	0.98 (0.89–1.06)

*Adjusted for the time-dependent variables age and childbirth (up to the year of observation, yes/no) and the fixed variables profession and country of birth. Adjustment for year of follow-up is inherent in the analytical model.

†Adjustment for night work ever was made to disentangle the effect of working night as such and the quantity of night work, see the Methods section.

**Table 3:** Risk of premenopausal and postmenopausal breast cancer in association with various aspects of night work in six separate models

	Premenopausal women				Postmenopausal women			
	Person-years	Cases	Crude HR	Adjusted HR*	Person-years	Cases	Crude HR	Adjusted HR*
No night work	72 438	108	1	1	34 765	108	1	1
Night work ever	41 851	39	0.63 (0.43–0.91)	0.72 (0.49–1.04)	11 146	44	1.26 (0.88–1.77)	1.31 (0.91–1.85)
No night work	72 438	108	1	1	34 765	108	1	1
1–3 years night work	30 303	29	0.65 (0.42–0.97)	0.81 (0.52–1.20)	6 169	28	1.50 (0.97–2.25)	1.59 (1.02–2.38)
4–7 years night work	11 095	10	0.60 (0.29–1.11)	0.57 (0.27–1.05)	4 612	11	0.72 (0.36–1.29)	0.74 (0.37–1.33)
8+ years night work	453	.	0.00 (0.00–4.25)	0.00 (0.00–3.07)	365	5	4.49 (1.51–10.91)	4.33 (1.45–10.57)
No night work	72 438	108	1	1	34 765	108	1	1
Risk per year of night work	41 851	39	0.90 (0.79–1.00)	0.91 (0.80–1.01)	11 146	44	1.04 (0.95–1.12)	1.04 (0.95–1.13)
No night work	72 438	108	1	1	34 765	108	1	1
Night work ever†	41 851	39	0.60 (0.31–1.10)	0.86 (0.45–1.60)	11 146	44	1.41 (0.73–2.60)	1.53 (0.79–2.83)
Risk per year of night work			1.02 (0.84–1.23)	0.94 (0.77–1.13)			0.97 (0.83–1.13)	0.96 (0.82–1.11)
No night work	72 438	108	1	1	34 765	108	1	1
Risk per 100 nights	41 851	39	0.94 (0.81–1.07)	0.92 (0.79–1.03)	11 146	44	1.01 (0.92–1.09)	1.01 (0.92–1.09)
No night work	72 438	108	1	1	34 765	108	1	1
Night work ever†	41 851	39	0.60 (0.38–0.92)	0.77 (0.48–1.19)	11 146	44	1.46 (0.91–2.26)	1.52 (0.95–2.37)
Risk per 100 nights			1.04 (0.88–1.19)	0.97 (0.82–1.11)			0.95 (0.84–1.06)	0.95 (0.84–1.05)

*Adjusted for the time-dependent variables age and childbirth (up to the year of observation, yes/no), and the fixed variables profession and country of birth. Adjustment for year of follow-up is inherent in the analytical model.

†Adjustment for night work ever was made to disentangle the effect of working night as such and the quantity of night work, see the Methods section.

The risk of breast cancer was subdivided according to menopausal state (table 3). In post-menopausal women, the adjusted HR associated with night work ever was non-significantly increased, HR 1.31 (95% CI 0.91 to 1.85). A subdivision according to number of years with night work showed an increased risk after 8 years or more with night work, adjusted HR 4.33 (95% CI 1.45 to 10.57), based on five cases only. Analysis of risk in association with number of years with night work or total number of nights (included in the model as continuous variables) showed no evidence of exposure-response for any of these metrics. Inclusion of indicators for night work ever did not change these results. In premenopausal women, there was no evidence for an increased risk in association with night work. It should be noted, though, that no women with premenopausal breast cancer had worked at night for 8 years or more.

## Discussion

The conclusions from this study are limited by a short period of follow-up and lack of information on night work before 2008. There was an increased risk of postmenopausal breast cancer in healthcare workers in Stockholm after 8 or more years of work that included nights shifts. The risk estimate was statistically significantly elevated but based on five cases only and must be interpreted with caution. There was no evidence of increased risk after shorter periods of work, and analyses of dose response in terms of number of years of night work or total number of nights worked showed no statistically significant trends. No elevated risks were found among premenopausal women, but there were no women with breast cancer who had worked at night for at least 8 years in this group. In the entire cohort, the adjusted risk estimate in association with 8 years of night work or more was non-significantly elevated, HR 2.80 (95% CI 0.96 to 6.52).

The current finding gives some weak support to previous studies indicating an association between night work and risk of breast cancer.^3^ The increased risk of postmenopausal breast cancer is opposed to the findings in some but not all earlier studies. A meta-analysis of five population-based case–control studies showed an increased risk predominantly in premenopausal women.^14^ A recent study from Finland found an association in postmenopausal but not in premenopausal women, though.^7^ Differences between studies in duration of exposure, timing of exposure and follow-up time in premenopausal and postmenopausal women may contribute to the variable findings in this respect.

This study indicated an increased risk after 8 years of night work or more. However, the number of years of night work may have been underestimated in this study, since information on night work before 2008 was not available. Some of the earlier studies of nurses have shown an increased risk first after about 20 years of exposure or more, for example, the large Nurses’ Health Study from the USA^15^ and a study of Norwegian nurses.^16^ However, the meta-analysis of five population-based case–control studies showed an increased risk also after <10 years of exposure in premenopausal women who had worked >3 nights per week.^14^ A study of Danish nurses showed an increased risk among those who had worked at night for 5–10 years, although the risk was even higher in those working >10 years.^17^ Thus, our finding of an increased risk after 8 or more years of night work is not contradictory to earlier findings, but as said above, we do not know the distribution of number of years of night work in this group.

This study has a strength in the detailed and record-based data on night work. Several previous findings, especially from case–control studies, have been criticised for potential problems in the exposure assessment, and the findings have not been replicated in cohort studies.^3 5^ The present cohort study, with detailed exposure data from records, has no potential for so-called recall bias, and the recent cohort study from Finland also shares this characteristic.^6^ Our study also has an advantage in that the risk estimates were adjusted for childbirths. This adjustment is important since there was a negative association between night work and previous childbirths which, if not adjusted for, could contribute to a false association between night work and breast cancer risk.

The study also has limitations. First, we had no information on employment and night work before 2008, even if some of the cohort members may have worked at nights during that period. This means that the number of night years may have been underestimated for some of the cohort members. The category of 8 or more years of night work, in which we found an excess of breast cancer is an open class, and some of the women may have worked at night for longer times. This does not affect the magnitude of the HR for this group, though. The second limitation is the relatively short period of follow-up.

We have very detailed data on how many nights each subject had worked for the period 2008–2016, but a subdivision with regard to number of nights per time unit was not possible in view of the small number of cases in the 8+ years group. The fact that we found an excess risk in postmenopausal women after 8 years involving nights, but no significant trend in terms of number of night years may seem contradictory. However, the five cases in the highest category have a small influence in the trend test in view of the much larger groups with shorter durations of night work.

## General conclusions

The conclusions from this study are limited due to a short follow-up period, a low number of cases with long-term night work, and lack of work histories before 2008. However, an excess risk of breast cancer was found in the longest available category of duration (8+ years), but this observation was based on five cases only and must be interpreted with caution. The study gives some weak support to earlier observations of an elevated risk of breast cancer in women in association with night work. The concentration of risk to postmenopausal breast cancer is in opposition to some but not all earlier findings. Further analysis of risk in relation to the quantities of night work per time unit was not possible due to low numbers. A prolonged follow-up is necessary for more precise conclusions from this cohort regarding the risk of breast cancer in association with night work.

## Data Availability

Data may be obtained from a third party and are not publicly available. Primary data can not be shared from reasons of confidentiality.
